# Detection of a Novel Alphaherpesvirus and Avihepadnavirus in a Plantar Papilloma from a Rainbow Lorikeet (*Trichoglosis moluccanus*)

**DOI:** 10.3390/v15102106

**Published:** 2023-10-17

**Authors:** Subir Sarker, David N. Phalen

**Affiliations:** 1 Biomedical Sciences & Molecular Biology, College of Public Health, Medical and Veterinary Sciences, James Cook University, Townsville, QLD 4811, Australia; 2 Sydney School of Veterinary Science, The University of Sydney, Camden, NSW 2570, Australia; 3 Schubot Exotic Bird Health, Texas A&M College of Veterinary Medicine and Biomedical Sciences, College Station, TX 77843-4467, USA

**Keywords:** parrots, papilloma, alphaherpesvirus, avihepadnavirus, evolution

## Abstract

Cutaneous plantar papillomas are a relatively common lesion of wild psittacine birds in Australia. Next-generation sequencing technology was used to investigate the potential aetiologic agent(s) for a plantar cutaneous papilloma in a wild rainbow lorikeet (*Trichoglosis moluccanus*). In the DNA from this lesion, two novel viral sequences were detected. The first was the partial sequence of a herpesvirus with the proposed name, psittacid alphaherpesvirus 6, from the *Mardivirus* genus of the family alphaherpesviruses. This represents the first mardivirus to be detected in a psittacine bird, the first mardivirus to be detected in a wild bird in Australia, and the second mardivirus to be found in a biopsy of an avian cutaneous papilloma. The second virus sequence was a complete sequence of a hepadnavirus, proposed as parrot hepatitis B genotype H (PHBV-H). PHBV-H is the first hepadnavirus to be detected in a wild psittacine bird in Australia. Whether other similar viruses are circulating in wild birds in Australia and whether either of these viruses play a role in the development of the plantar papilloma will require testing of biopsies from similar lesions and normal skin from other wild psittacine birds.

## 1. Introduction

Herpesvirus infections are widespread in birds raised for food, including poultry, domestic ducks, and rock pigeons. They are also widespread in many free-ranging wild birds and wild birds kept in captivity. All avian herpesviruses are in the alphaherpesvirus family and are either members of the genera *Mardivirus* or *Iltovirus* [1]. All herpesviruses detected in psittacine birds (parrots and their allies) are Itoviruses [2,3,4,5,6,7]. Mucosal [4] and cutaneous [2,4] papillomas are well documented lesions in wild-caught and domestically raised Psittacine birds held in North American and European collections. Mucosal papillomas of the digestive system contain and are likely caused by psittacid alphaherpesvirus 1, genotypes 1, 2, and 3 [8]. These lesions are most commonly found in neotropical species of psittacine birds but are infrequently reported in psittacine birds from the Indopacific distribution [4]. Mucosal papillomas, sometimes extending to the skin of the face and containing psittacid herpesvirus 2 DNA, are also documented in wild-caught and captive-raised Congo African grey parrots (*Psitticus erithacus erithacus*) [2,4]. Another member of the iltoviruses, the *Phoenicapterid alphaherpesvirus*, was detected in plantar lesions of an unrelated species, the Greater Flamingo (*Phoenicopterus roseus*). Some of these lesions were characterised microscopically as being composed of papillary changes [9]. Two mardiviruses, the Fregata magnificens alphaherpesvirus and the Onyxhopeion fucatus alphaherpesvirus, have been detected in some but not all hyperkeratotic skin lesions in magnificent frigatebirds (*Fregata magnificens*) [10] and sooty terns (*Onychoprion fuscatus*), respectively [11]. Whether these mardiviruses were incidentally present in the lesions of these species is not known.

Hepadnaviruses have been detected in both wild and captive birds. In wild birds, hepadnaviruses have been found in several species of geese and ducks, and a single species of heron, and a single species of crane [12]. In captive birds, hepadnaviruses have been detected in multiple species of parrot and in elegant-crested tinamous (*Eudromia elegans*) [13]. From these studies, it is known that all hepadnaviruses that infect birds are members of the *Avihepadnavirus* genus, are hepatotrophic, and appear, in general, to have a limited host range. Additionally, based on studies of the duck hepatitis B virus, avian hepadenoviruses appear to be transmitted vertically and horizontally, and depending on the age of the bird at the time of infection, infections can be persistent (reviewed in Funk et al. [14]). Infection with avihepadnaviruses is rarely associated with disease [12], although a chronic active hepatitis was identified in multiple elegant-crested tinamous (*Eudromia elegans*) infected with a novel avihepadnavirus in a collection in Germany [13]. Whether this was a host-adapted virus of elegant-crested tinamous or a virus originating from another species of bird is not known, as infection was not found in elegant-crested tinamous in other collections. Also, unlike Orthohepadnaviruses, which infect mammals, infection with members of the *Avihepadnavirus* genus is not associated with the development of hepatic carcinomas in their host species (reviewed in Funk et al. [14]).

There are seven known parrot hepatitis B genotypes (PHBV) (A-G), which were identified in a survey for avihepadnaviruses in captive parrots submitted for necropsy in Poland [15]. All genotypes, but D, were detected in Indian ringneck parakeets *(Psittacula krameria*). Genotype D was only detected in an Alexandrine parakeet (*Psittacula eupatria*). Genotype B was also found to infect a Congo African Grey Parrot, and genotype G was also detected in a species of Australian origin, a crimson rosella (*Platycercus elegans*). The species of origin of these PHBV virus genotypes is not known, as opportunities for cross-species infections would be high, as most captive Psittaformes are kept in mixed collections of parrots. However, the discovery of a full-length endogenous PHVB in budgerigars suggests that the ancestors of today’s PHVB viruses circulated in wild budgerigars (*Melopsittacus undulatus*) at least 2.5 mya and possibly more than 5 mya [16]. Whether the PHBV genotypes can cause disease is not known. Many were detected in birds with hepatitis, but these birds were also infected with either the Budgerigar fledgling disease virus or the psittacine beak and feather disease virus or both as both of these viruses can cause hepatitis, and, therefore, it is not known if concurrent infection with PHBV played any role in the observed lesions [1].

In this paper, we present the partial genome sequence of a novel alphaherpesvirus and the complete sequence of a novel avihepadnavirus that were detected in a cutaneous papilloma from the foot of a wild rainbow lorikeet (*Thricoglossus moluccanus*).

## 2. Materials and Methods

### 2.1. Sampling, DNA Extraction and Sequencing

Tissue scrapings from cutaneous papillomatous lesions of an adult rainbow lorikeet were collected using a sterile scalpel blade with the bird under isoflurane anaesthesia. The bird was presented to the University of Sydney, Sydney School of Veterinary Science, Avian Reptile and Exotic Pet Hospital because it was unable to fly. It was anaesthetized for radiography to determine if it had skeletal injuries when the cutaneous lesions were observed. Total genomic DNA was extracted using a commercial extraction kit (DNeasy Blood & Tissue Kit, Qiagen, Doncaster, Victoria, Australia) according to the manufacturer’s directions. The library construction was adapted using the Nextera DNA Flex Prep (Illumina, San Diego, CA, USA) as per kit instructions [17]. The quality and quantity of the prepared library were assessed (AGRF, Westmead, NSW, Australia). The prepared library was normalised at a picomole concentration as per Illumina DNA library prep instructions. The quality and quantity of the final library was further assessed before sequencing by the AGRF facility. Cluster generation and sequencing of the library was performed with the read length of 150 bp paired end on Illumina^®^ HiSeq chemistry according to the manufacturer’s instructions.

### 2.2. Sequence Data Analysis

The resulting raw sequencing reads were analysed as per the established pipeline [18] using Geneious Prime^®^ (version 2022.1.1, Biomatters, Auckland, New Zealand) and the CLC Genomics Workbench (version 9.0.1, CLC bio, a QIAGEN Company, Prismet, Aarhus C, Denmark). Briefly, a preliminary quality evaluation for all raw reads was generated and pre-processed to remove ambiguous base calls and poor-quality reads and trimmed to remove the Illumina adapter sequences. Trimmed sequence reads were mapped against the chicken genome (GenBank accession number NC_006088.5) to remove likely host DNA contamination. In addition, reads were further mapped to the *Escherichia coli* bacterial genomic sequence (GenBank accession no. U00096) to remove possible bacterial contamination. Unmapped reads were subjected to de novo assembly using SPAdes assembler (version 3.10.1) [19], under the “careful” parameter in the LIMS-HPC cluster (La Trobe Institute for Molecular Science—High Performance Computing cluster, specialised for genomics research at La Trobe University) [20,21]. Resulting contigs were compared against the nonredundant nucleotide and protein databases on GenBank using BLASTn and BLASTx [22], respectively, with an e-value threshold of 1 × 10^−5^ to remove potential false positives. BLASTN searches yielded two contigs of 103,445 and 3690 bp corresponding to a partial novel alphaherpesvirus (psittacid alphaherpesvirus 6 (PsAHV6), average coverage of 4126.51x) and a complete avihepadnavirus (parrot hepatitis B virus (PHBV), average coverage of 39.76x), respectively.

### 2.3. Genome Annotation and Bioinformatics

The assembled viral genomes were annotated using Geneious Prime^®^ (version 2022.1.1, Biomatters, Ltd., Auckland, New Zealand), with gallid alphaherpesvirus 2 (GaAHV2, GenBank accession no. KU744558.1) [23] and parrot hepatitis B virus (PHBV, GenBank accession no. JX274033.1) [15] used as reference genomes for PsAHV6 and PHBV, respectively. In the case of PsAHV6, several avian alphaherpesvirus genomes were used as references for the annotation process to compare the protein sequences of predicted ORFs and to evaluate the consequences of potential truncations or extensions that can occur at the N- and C-termini of predicted proteins and orthologues. ORFs over 50 amino acids along with minimal overlapping (not exceeding 25% overlaps in one of the genes) to other open reading frames were selected and annotated. The predicted ORFs for both genomes were extracted into FASTA files subsequently, and similarity searches were performed on annotated ORFs as potential genes to determine whether they shared significant sequence similarities to established viral or cellular genes (BLAST E value ≤ 10^−5^) or contained a putative conserved domain as predicted by protein searches (BLASTX and BLASTP) [22].

To predict the function of predicted hypothetical proteins, multiple applications were used to search the derived protein sequence of each ORF and identify their conserved domains or motifs. TMHMM package v.2.0 (DTU Health Tech, Lyngby, Denmark) [24], Geneious Prime^®^ (version 2022.1.1), HMMTOP [25], and TMpred [26] were used to search transmembrane (TM) helices. Conserved secondary structure (HHpred) [27] and protein homologs were searched using Phyre2 [28] and SWISS-MODEL [29] to help predict the function of predicted ORFs in this study.

### 2.4. Phylogenetic Analyses

Sequence similarity percentages between representative viruses were determined using tools available in Geneious Prime^®^ (version 2022.1.1). For phylogenetic analysis, demonstrative herpesvirus and avihepadnavirus gene sequences were downloaded from GenBank, and trees were constructed using Geneious Prime^®^ (version 2022.1.1). The amino acid sequences of protein-coding genes from the respective viruses were aligned using the MAFTT L-INS-I algorithm (scoring matrix BLOSUM62; gap open penalty 1.53; off set value 0.123) (version 7.388) [30] implemented in Geneious Prime^®^ (version 2022.1.1). The amino acid sequences of alphaherpesviruses (*n* = 25) of complete DNA polymerase genes except for Falco tinnunculus alphaherpesvirus 1 and Aquila chrysaetos alphaherpesvirus 1, for which only partial DNA polymerase sequences were chosen for phylogenetic analysis. For the phylogenetic analysis of avihepadnaviruses, amino acid sequences of a total of 24 complete DNA polymerase genes (approximate length between 549 and 843 residues) were selected. Phylogenetic analysis was performed using the LG substitution model with 1000 bootstrap replicates in Geneious Prime^®^ (version 2022.1.1).

## 3. Results

### 3.1. Evidence of a Novel Psittacid Alphaherpesvirus 6

The partial genome of a psittacid alphaherpesvirus 6 (PsAHV6) detected in this study was a linear dsDNA molecule of 103,445 base pairs, with an average coverage of 4126.51x and a G + C content of 47.7%. We were able to sequence approximately two-thirds of the genome of PsAHV6, which lacks UL01 to UL19 genes (Table 1). The PsAHV6 partial genome had 69 predicted methionine-initiated open reading frames (ORFs) encoding proteins that were annotated as putative genes and were numbered from left to right (Table 1). Comparative analysis of the protein sequences encoded by the predicted ORFs, using BLASTX and BLASTP, identified homologs with significant protein sequence similarity for 40 ORFs (Table 1). Among these conserved herpesvirus gene products, the highest number of protein-coding genes (20) in PsAHV6 demonstrated homologs to the previously sequenced falconid herpesvirus 1 (FaAHV1). The remaining 10 gene products of PsAHV6 (ORF001, -008, -014, -018, -021, -029, -037, 056, -057, and -061) were homologous to ORFs of Columbid alphaherpesvirus 1 (CoAHV1), and a further six gene products (ORF011, -016, -031, -034, -050, and -062) were homologs to gallid alphaherpesvirus 2 (GaAHV2), two were (ORF007 and -010) homologs to anatid alphaherpesvirus (AnAHV1), and two were (ORF012 and -027) homologs to meleagrid alphaherpesvirus 1 (MeAHV1) (Table 1). Among the 40 homologous protein-coding genes of PsAHV6, the amino acid identities were relatively low, in the range of 28.14% to 67.17% (Table 1).

PsAHV6 contained 29 predicted protein-coding genes that were not present in any other herpesvirus, nor did they match any sequences in the NR protein database using BLAST search; these unique ORFs encoded proteins of 25-452 aa in length (Table 1).

### 3.2. Evolutionary Relationship of PsAHV6

**Figure 1:**
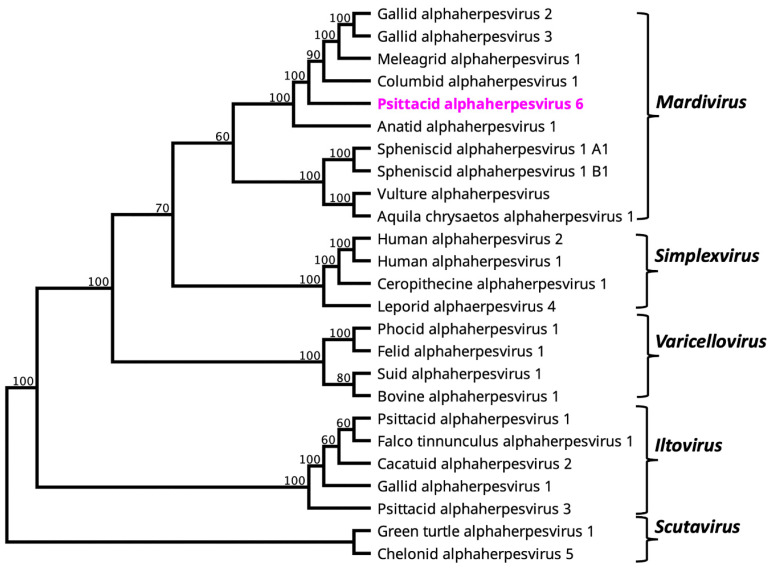
A maximum likelihood phylogenetic tree was constructed from the amino acid sequences of selected alphaherpesviruses using complete DNA polymerase genes except for Falco tinnunculus alphaherpesvirus 1 and Aquila chrysaetos alphaherpesvirus 1, for which only partial DNA polymerase sequences available. The numbers on the left show bootstrap values as percentages. The labels at branch tips refer to virus name. The novel PsAHV6 from rainbow lorikeet is shown in pink colour.

The DNA polymerase sequences of the selected alphaherpesviruses, which all shared between 42.11 and 61.50% amino acids similarity to the PsAHV6 sequence were detected in rainbow lorikeet. The generated phylogram using the same set of sequences for which there were full DNA polymerase sequences available (Figure 1) evidenced the first *Mardivirus,* psittacine alphaherpesvirus 6 (PsAHV6), that infects psittacine birds. The PsAHV6 was clustered within clade A of the *Mardivirus* genus, which also contains the originally sequenced Mardiviruses, including the Marek’s disease virus for which this genus was named, the columbid alphaherpesvirus, and the anatid alphaherpesvirus 1. In clade A of the *Mardivirus* genus, anatid alphaherpesvirus 1 is basal to known avian alphaherpesviruses, suggesting that all the alphaherpesviruses within this clade evolved from the ancestral domestic duck (*Anas platyrhynchos*), from where PsAHV6, followed by other avian alphaherpesviruses in this clade, evolved (Figure 1).

### 3.3. Evidence of a Novel Avihepadnavirus

In this study, a novel complete genome of parrot hepatitis B virus (PHBV) is reported (GenBank accession number, ON688522; average coverage of 39.76x) and determined to be 3690 bp in length (G + C content, 49.2%). This is the first reported genome of PHBV that has been sequenced from a wild Australian psittacine bird. The complete genome of PHBV has a similar genomic organisation to other avihepadnaviruses, with three main open reading frames encoding the PreC/C, PreS/S, and polymerase polyproteins. The PHBV genome determined here showed the highest sequence similarity of 88.43% (query coverage of 62%) with the parrot parrot hepatitis B virus (GenBank accession no. JX274033.1) sequenced from ringnecked parakeets in Poland [15].

**Figure 2:**
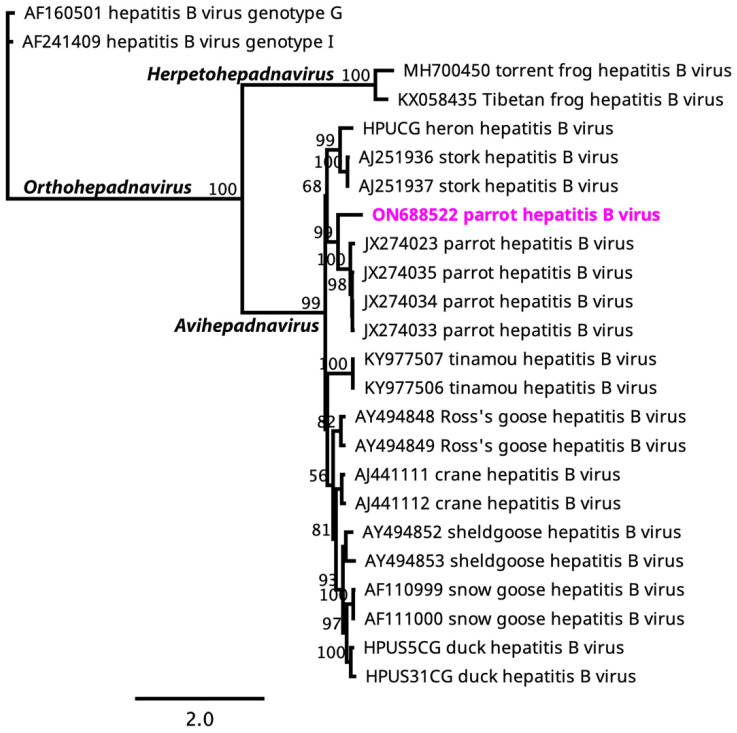
An unrooted maximum likelihood phylogenetic tree was constructed from the amino acid sequences of selected avihepadnaviruses using complete DNA polymerase genes. The numbers on the left show bootstrap values as percentages. The labels at branch tips refer to GenBank accession numbers, followed by virus name. The novel parrot hepatitis B virus from rainbow lorikeet is shown in pink colour.

A maximum likelihood (ML) phylogenetic tree of the DNA polymerase gene of the selected avihepadnaviruses (Figure 2) indicated that the novel PHBV clustered within the clade dominated by parrot hepatitis B virus. Importantly, PHBV sequenced from this study is basal to other parrot hepatitis B viruses, suggesting that PHBV in this clade likely evolved from the ancestral rainbow lorikeet (*Trichoglossus moluccanus*) (Figure 2).

## 4. Discussion

In this report, we describe the partial and complete genomes of a novel alphaherpesvirus and a novel avihepadnavirus, respectively, detected in a papillomatous lesion biopsied from the foot of a wild rainbow lorikeet. The novel herpesvirus, proposed name psittacid alphaherpesvirus-6 (PsAHV6), is a mardiherpesvirus and is the first mardivirus found in a captive or wild psittacine bird and the first mardivirus detected in a wild bird in Australia. Whether this proposed PsAHV6 represents a mardivirus that has undergone a host switch or is the first of a novel set of herpesviruses that have evolved in Australian psittacine birds remains to be determined, screening similar cutaneous lesions and biopsies of normal skin in other wild Australian psittacine birds for PsAHV6 DNA or DNA from other herpesviruses may prove useful in addressing this question.

Whether PsAHV6 was an incidental finding in the biopsy of the plantar papilloma of this rainbow lorikeet or was an aetiological agent of this lesion is not known. The Iltoviruses, psittacid alphaherpesvirus 1 (genotypes 1, 2, and 3) and psittacid alphaherpesvirus 2 have been associated with and appear to be the aetiologic agent of mucosal and cutaneous papillomas in psittacine birds [4,8]. Additionally, a mardivirus has been detected in cutaneous papillomas on the plantar surface of the feet of greater flamingos [9]. If PsAHV6 has the same potential to cause papillomas, these other alphaherpesviruses will require additional investigation, including screening additional plantar lesions of wild psittacine birds for this PsAHV6 and localizing the presence of this virus within the lesion using an in situ hybridization assay. Given the increasing number of whole genomes of alphaherpesviruses found in avian papillomatous lesions and those that are not, it may be possible to use in silico methods to identify conserved genes that could predispose to these lesions.

Hepadnaviruses have been previously shown to infect Anseriformes (ducks and geese) [31], Pelicaniformes (herons), and Gruiformes (cranes) in captive collections and in the wild [14]. The detection of an endogenous hepadnavirus in budgerigars (Liu et al., 2012) [16], an Australian species, and a study of captive psittacine birds in Poland that were submitted for necropsy [15] suggest that hepadnavirus infection may also occur in wild psittaciformes as well. Our detection of a hepadnavirus in a wild rainbow lorikeet represents the first detection of a hepadnavirus in a wild psittaformes and the first detection of a hepadnavirus in a wild bird in Australia. Given that its sequence and the sequence of the endogenous budgerigar hepadnavirus are similar to those detected so far in other species of parrot, this suggests that parrot hepadnaviruses might first have evolved in wild psittacine birds in Australia, but additional testing of other wild Australian psittacine birds would be required to confirm this hypothesis.

The human hepatitis B virus, an orthohepadnavirus, is known to be the cause of liver cancer in humans [14]. While some avian hepadnaviruses cause liver disease in birds [13,15], a correlation between avian hepadnavirus and neoplasia development in their host has not been documented. Therefore, it is unlikely that PsAHV6 was directly involved in the pathogenesis of the plantar papilloma in which it was detected. Instead, it would seem more likely that PsAHV6 was present in the lorikeet blood and was an incidental finding. Viremia has been shown to be common in birds infected with avian hepadnaviruses [13]. Screening serum and papillomatous lesions as well as the normal skin of affected and unaffected wild Australian psittacine birds would be necessary to test this hypothesis.

## Figures and Tables

**Table 1:** Psittacid alphaherpesvirus 6 (PsAHV6) genome annotation (partial) and comparative analysis of ORFs.

PsAHV6 Synteny	PsAHV6 Gene Coordinate	Nt Length	AA Length	Best Hit Gene Product	Best Hit (%Identity/Query Coverage/e-Value/PI/Organism)
PsAHV6-001	1128-301	828	275	envelope protein UL20	38.43/85/6.00E-36/YP_009352926.1/CoAHV1
PsAHV6-002	1628-1885	258	85	hypothetical protein	no significant BLAST hits
PsAHV6-003	1959-3761	1803	600	tegument protein UL21	33.28/92/7.00E-89/YP_009046517.1/FaAHV1
PsAHV6-004	3847-4176	330	109	hypothetical protein	no significant BLAST hits
PsAHV6-005	6679-4010	2670	889	envelope glycoprotein H UL22	33.87/88/1.00E-120/YP_009046519.1/FaAHV1
PsAHV6-006	8196-7066	1131	376	thymidine kinase UL23	36.47/85/7.00E-59/YP_009046521.1/FaAHV1
PsAHV6-007	8112-9194	1083	360	nuclear protein UL24	35.27/66/4.00E-26/ABK55352.1/AnAHV1
PsAHV6-008	9374-11428	2055	684	DNA packaging tegument protein UL25	54.55/82/5.00E-155/YP_009352933.1/CoAHV1
PsAHV6-009	11370-11570	201	66	hypothetical protein	no significant BLAST hits
PsAHV6-010	11752-14400	2649	882	capsid maturation protease UL26	56.57/28/2.00E-73/UJO49828.1/AnAHV1
PsAHV6-011	17814-14731	3084	1027	envelope glycoprotein B UL2	60.91/80/0/CAA63039.1/GaAHV2
PsAHV6-012	20681-17586	3096	1031	DNA packaging terminase subunit 2 UL28	51.93/71/1.00E-151/NP_073322.1/MeAHV1
PsAHV6-013	20801-21118	318	105	hypothetical protein	no significant BLAST hits
PsAHV6-014	25126-21098	4029	1342	single-stranded DNA-binding protein UL29	51.90/99/0/YP_009352937.1/CoAHV1
PsAHV6-015	25389-25129	261	86	hypothetical protein	no significant BLAST hits
PsAHV6-016	25677-29612	3936	1311	DNA polymerase UL30	54.06/98/0/UOW62139.1/GaAHV2
PsAHV6-017	30888-29536	1353	450	nuclear egress lamina protein UL31	58.70/67/6.00E-111/YP_009046529.1/FaAHV1
PsAHV6-018	33797-31323	2475	824	DNA packaging protein UL32	51.12/84/2.00E-126/YP_009352940.1/CoAHV1
PsAHV6-019	33808-34290	483	160	DNA packaging protein UL33	56.49/81/2.00E-34/YP_009046531.1/FaAHV1
PsAHV6-020	34360-34557	198	65	hypothetical protein	no significant BLAST hits
PsAHV6-021	34643-35719	1077	358	nuclear egress membrane protein UL34	58.62/48/5.00E-66/YP_009352942.1/CoAHV1
PsAHV6-022	35815-36162	348	115	small capsid protein UL35	47.92/81/9.00E-12/YP_009046533.1/FaAHV1
PsAHV6-023	46419-36400	10020	3339	large tegument protein UL36	37.71/71/0/YP_009046534.1/FaAHV1
PsAHV6-024	46742-46416	327	108	hypothetical protein	no significant BLAST hits
PsAHV6-025	50376-46966	3411	1136	tegument protein UL37	36.23/98/0/YP_009046535.1/FaAHV1
PsAHV6-026	50892-52397	1506	501	capsid triplex subunit 1 UL38	54.68/93/1.00E-144/YP_009046536.1/FaAHV1
PsAHV6-027	52766-55537	2772	923	ribonucleotide reductase subunit 1 UL39	56.43/86/0/NP_073333.1/MeAHV1
PsAHV6-028	56022-57398	1377	458	ribonucleotide reductase subunit 2 UL40	67.17/71/1.00E-156/YP_009046538.1/FaAHV1
PsAHV6-029	59232-57688	1545	514	tegument host shutoff protein UL41	44.74/73/3.00E-79/YP_009352949.1/CoAHV1
PsAHV6-030	59365-59556	192	63	hypothetical protein	no significant BLAST hits
PsAHV6-031	60266-61675	1410	469	DNA polymerase processivity subunit UL42	42.02/73/5.00E-84/AUB50956.1/GaAHV2
PsAHV6-032	62162-63520	1359	452	hypothetical protein	no significant BLAST hits
PsAHV6-033	64113-63922	192	63	hypothetical protein	no significant BLAST hits
PsAHV6-034	64380-66293	1914	637	glycoprotein C UL44	34.00/62/3.00E-78/AAM97710.1/GaAHV2
PsAHV6-035	67702-66737	966	321	hypothetical protein FaHV1S18_060	34.68/90/5.00E-45/YP_009046544.1/FaAHV1
PsAHV6-036	68228-68416	189	62	hypothetical protein	no significant BLAST hits
PsAHV6-037	68425-69114	690	229	membrane protein UL45	40.85/71/1.00E-40/YP_009352955.1/CoAHV1
PsAHV6-038	72032-69492	2541	846	tegument protein VP11/12 UL46	28.57/47/4.00E-40/YP_009046546.1/FaAHV1
PsAHV6-039	71997-72278	282	93	hypothetical protein	no significant BLAST hits
PsAHV6-040	72552-72283	270	89	hypothetical protein	no significant BLAST hits
PsAHV6-041	72854-73048	195	64	hypothetical protein	no significant BLAST hits
PsAHV6-042	75190-73202	1989	662	tegument protein VP13/14 UL47	33.16/81/3.00E-67/YP_009046547.1/FaAHV1
PsAHV6-043	75135-75587	453	150	hypothetical protein	no significant BLAST hits
PsAHV6-044	75720-75475	246	81	hypothetical protein	no significant BLAST hits
PsAHV6-045	76074-75808	267	88	hypothetical protein	no significant BLAST hits
PsAHV6-046	76919-76722	198	65	hypothetical protein	no significant BLAST hits
PsAHV6-047	78218-77028	1191	396	transactivating tegument protein VP16 UL48	45.56/89/9.00E-95/YP_009046548.1/FaAHV1
PsAHV6-048	78299-78481	183	60	hypothetical protein	no significant BLAST hits
PsAHV6-049	78811-78533	279	92	hypothetical protein	no significant BLAST hits
PsAHV6-050	79856-79092	765	254	tegument protein VP22 UL49	38.36/28/7.00E-07/UOW62334.1/GaAHV2
PsAHV6-051	79855-80055	201	66	hypothetical protein	no significant BLAST hits
PsAHV6-052	80468-80181	288	95	envelope glycoprotein N UL49.5	51.04/98/8.00E-11/YP_009046550.1/FaAHV1
PsAHV6-053	81124-82521	1398	465	deoxyuridine triphosphatase UL50	40.74/98/2.00E-103/YP_009046551.1/FaAHV1
PsAHV6-054	82780-82541	240	79	hypothetical protein	no significant BLAST hits
PsAHV6-055	83633-82671	963	320	tegument protein UL51	52.58/60/7.00E-59/YP_009046552.1/FaAHV1
PsAHV6-056	83632-87303	3672	1223	helicase-primase primase subunit UL52	42.05/94/0/YP_009352963.1/CoAHV1
PsAHV6-057	87325-88494	1170	389	envelope glycoprotein K UL53	49.29/89/2.00E-98/YP_009352964.1/CoAHV1
PsAHV6-058	88959-90398	1440	479	multifunctional expression regulator UL54	37.67/44/8.00E-30/YP_009046555.1/FaAHV1
PsAHV6-059	91648-90716	933	310	protein LORF4	36.59/87/3.00E-50/YP_009046556.1/FaAHV1
PsAHV6-060	92305-92919	615	204	nuclear protein UL55	46.43/81/6.00E-46/YP_009352967.1/FaAHV1
PsAHV6-061	94339-93110	1230	409	myristylated tegument protein CIRC	33.19/55/5.00E-13/YP_009352969.1/CoAHV1
PsAHV6-062	97772-94611	3162	1053	hypothetical protein LORF11	28.14/86/4.00E-81/UOW65035.1/GaAHV2
PsAHV6-063	99543-98449	1095	364	hypothetical protein	no significant BLAST hits
PsAHV6-064	99943-100107	165	54	hypothetical protein	no significant BLAST hits
PsAHV6-065	100621-100463	159	52	hypothetical protein	no significant BLAST hits
PsAHV6-066	101828-100758	1071	356	hypothetical protein	no significant BLAST hits
PsAHV6-067	102213-101770	444	147	hypothetical protein	no significant BLAST hits
PsAHV6-068	102515-102832	318	105	hypothetical protein	no significant BLAST hits
PsAHV6-069	103442-103113	330	109	hypothetical protein	no significant BLAST hits

Note: %, percentage, PI, Protein Identifier sequence identification number sourced by BLAST searches. The abbreviations for herpesviruses were used: PsAHV6, psittacid alphaherpesvirus 6; CoAHV1, columbid alphaherpesvirus 1; FaAHV1, falconid herpesvirus 1; GaAHV2, gallid alphaherpesvirus 2; AnAHV1, anatid alphaherpesvirus 1; MeAHV1, meleagrid alphaherpesvirus 1.

## Data Availability

The sequences and associated data analysed in this study have been deposited in NCBI GenBank under the accession numbers ON688521-ON688522.
